# Age at Nomination Among Soccer Players Nominated for Major International Individual Awards: A Better Proxy for the Age of Peak Individual Soccer Performance?

**DOI:** 10.3389/fpsyg.2021.661523

**Published:** 2021-05-24

**Authors:** Geir Oterhals, Håvard Lorås, Arve Vorland Pedersen

**Affiliations:** ^1^Faculty of Business Administration and Social Sciences, Molde University College, Molde, Norway; ^2^Department of Teacher Education, Faculty of Social and Educational Sciences, Norwegian University of Science and Technology, Trondheim, Norway; ^3^Department of Neuromedicine and Movement Science, Faculty of Medicine and Health Sciences, Norwegian University of Science and Technology, Trondheim, Norway

**Keywords:** football, performance, elite, team sport, optimal, career

## Abstract

Individual soccer performance is notoriously difficult to measure due to the many contributing sub-variables and the variety of contexts within which skills must be utilised. Furthermore, performance differs across rather specialised playing positions. In research, soccer performance is often measured using combinations of, or even single, sub-variables. All too often these variables have not been validated against actual performance. Another approach is the use of proxies. In sports research, the age of athletes when winning championship medals has been used as a proxy for determining their age of peak performance. In soccer, studies have used the average age of players in top European leagues or in the Champions League to determine the age of individual peak performance. Such approaches have methodological shortcomings and may underestimate the peak. We explore the use of a new proxy, the age at nomination for major individual awards, to determine the average age at peak individual soccer performance. A total of 1,981 players nominated for major awards from 1956 to 2019 were included, and a subset of 653 retired players was extracted, thus including players’ complete careers. Players’ average ages at nomination, at their first nomination, and at their last ever nomination were calculated, and differences across playing positions were calculated together with changes over time in the average age at peak. Based on our proxy, the age of individual peak soccer performance occurs around 27–28 years, varying across playing positions from 26 to 31 years. A player’s first peak, on average, seems to coincide with known peaks of physiological variables; their last-ever peak occurs long after physiological performance has started to decline, indicating that the decline can be compensated for by other variables. The peak age is higher than previously reported for soccer; however, it is similar to those in other team ball sports. The average age at peak performance has increased over time, especially in the last decade. Our approach of using proxies for unearthing information about hidden features of otherwise immeasurable complex performance appears to be viable, and such proxies may be used to validate sub-variables that measure complex behaviour.

## Introduction

In soccer, team performance is measured in the simplest way: the number of scored goals minus the number of conceded goals (IFAB, 2020). Accumulated performance over time is measured by the total points earned in winning or drawing matches (usually three points and one point, respectively) in a league in one season (see, for example, Premier League, 2020). Individual soccer performance is more difficult to measure and is not formally assessed since *who* scores the goals does not affect the result. However, an abundance of statistics is kept on individual players to rank their performances relative to one another (e.g., Whoscored.com, Transfermarkt.com, fbref.com). Furthermore, teams keep accounts of players’ performances using a variety of sub-variables that contribute to individual soccer performance and, ultimately, to team performance.

A goal being scored or conceded is often due to a chain of events, during which each involved player contributes to the result to varying degrees and in different ways. Sometimes a player’s involvement can be decisive and easily measured, such as in scoring a goal, assisting a goal, saving a penalty, or keeping a clean sheet in the case of the goalkeeper. Most often, however, each player’s contribution to each goal (whether scored or conceded) is more subtle and cannot be readily measured.

Individual soccer performance is dependent on a variety of physiological factors such as running speed, endurance, muscle strength (see Slimani and Nikolaidis, 2017), and agility (Young and Farrow, 2006). Soccer players also must possess a number of different ball skills (see Wilson et al., 2020). For example, a player must be able to pass the ball over a variety of distances, with different requirements of force and accuracy, within a changing environment that includes opposing players who are actively trying to intercept the passes or retain the ball. The player must master shooting, controlling the ball with many different body parts, dribbling, heading, and more, with each different action involving a quite different, yet specific, skillset. Furthermore, all these skills need to be adapted to situations involving a varying number of teammates as well as players from the opposing team. Such decisions need to be timed well, and decisions often need to be taken in a matter of milliseconds (Roca et al., 2020) or the opportunity may be missed, and the player(s) will need to start searching for other opportunities. Thus, players need rather advanced perceptual (Oppici et al., 2017; McGuckian et al., 2018) and decision-making skills (Romeas et al., 2016).

To make the measurement process even more complicated, the players on each team do not necessarily possess the same mix of skills, especially due to their playing different positions on the field: a team is composed of highly specialised players with different specialities (Di Salvo et al., 2007; Ade et al., 2016; Vigh-Larsen et al., 2018). For example, defenders more often intercept, clear, and cross the ball, whereas midfielders perform more dribbles and tackle more often, as do fullbacks (Taylor et al., 2004). Goalkeepers have a completely different skillset, including catching shots, making saves or blocking shots (including dives), defending crosses, deflecting or punching the ball, as well as quick distribution of the ball by hand and by foot (Ziv and Lidor, 2011). Furthermore, there are different physical demands across playing positions. Midfielders run longer distances and at higher intensities compared with central defenders and forwards in particular (Altavilla et al., 2018). Also, wide defenders (fullbacks) spend more time performing high-intensity work compared with central defenders (Di Salvo et al., 2007; Lago-Peñas et al., 2009). These different requirements seem to influence the average age of players in different positions on the field, with defensive players (defenders and especially goalkeepers) being older than midfielders and forwards (Bloomfield et al., 2005; For a more thorough description of the complexity of the game, see McHale et al., 2012).

Within a host of domains like arts, culture, and science, peak performances seem to vary somewhat randomly within an individual’s career (Liu et al., 2018). In sports, however, where physical attributes play a greater role, careers are less random. Albeit variable, athletes’ careers are, in essence, biphasic, consisting of a period of growth and development followed by a period of decline (Guillaume et al., 2011; Berthelot et al., 2012). Consequently, soccer players, for whom physical capacity in part determines performance, will inevitably experience a decrease in performance with age due to physiological decline. Exactly when this decline will occur, and by how much, is uncertain since it can be compensated for by superior ball skills, perceptual skills, and tactical skills, but it is possible to speculate based on the known peaks for various physiological variables and their decline with age.

Mean peak performance among elite sprint specialists is achieved at 25–26 years of age (Haugen et al., 2018). For soccer players, sprint times improve from 12 years of age until adulthood (Baumgart et al., 2018), and they begin declining after the age of 28 (Haugen et al., 2020). Sprint performance is also strongly related to jumping performance among soccer players (Emmonds et al., 2019) due to the strong power requirements in both tasks (Methenitis et al., 2016). Specialists in power production, such as elite athletes in Olympic weightlifting, demonstrate their peak performance at 26 years of age (Huebner and Perperoglou, 2019), which corresponds well with peak performance in vertical jumping and sprint performance among soccer players.

The age at peak endurance performance among specialised athletes generally increases with duration of the event, and performance in endurance events of long duration may peak as late as the mid- to late-30s (Longo et al., 2016). In soccer, endurance among top-level Czech Republic players (measured as maximal oxygen uptake) was similar for players aged 17–30 years, while it was significantly lower for players aged 30–39 years (Botek et al., 2016). Tønnessen et al. (2013) reported that players younger than 18 years old had higher VO^2^_max_ values than players aged 23–26 years. However, soccer endurance is also dependent on playing position. Tønnessen et al. (2013), for example, reported midfielders to have higher maximal oxygen uptakes than defenders, forwards, and goalkeepers.

The findings reported above, which are based on measurements of isolated physiological capacities such as power generation and endurance, seem to correspond well with the findings of Sal de Rellán-Guerra et al. (2019), which are based on real soccer match performance. These authors reported that professional soccer players above 30 years of age ran significantly shorter distances and completed fewer fast runs and sprints during matches compared with players below 30 years of age. Interestingly, the same authors reported that players’ ability to make successful passes improved after they turned 30 years old. These effects were observed in all positional roles except wide midfielders (Sal de Rellán-Guerra et al., 2019). Furthermore, Wilson et al. (2017) showed that the best players in matches were those with superior technical (ball) skills rather than athletic traits such as speed, agility, strength, and stamina. Superior ball skills might help explain why some players can maintain a high performance level in soccer well beyond the age at which physiological capacities have peaked.

A vast number of tests aim at measuring soccer skill performance (Ali, 2011). Several studies have identified correlations between field test and match performance. For example, Rampinini et al. (2007) identified a correlation between an incremental running field test and high-speed running in matches. Still, it is possible that high-speed running does not correlate with success for the team. Hoppe et al. (2015) correlated match running performance with success in the German Bundesliga and found that match running performance is not the only skill needed to achieve success for teams; technical and tactical skills as well as ball possession are also important. However, Ré et al. (2014) found that, in general, for young players, the measures of technical skills obtained outside of games did not correlate with performance in games. Carling (2013) also cast doubt on the importance of physical performance for team success. Also, a team’s playing formation seems to impact on high intensity running activity and technical aspects of performance (Bradley et al., 2011). Furthermore, playing style affects match outcome (Castellano and Pic, 2019), and match status affects style of play (Fernandez-Navarro et al., 2018), illustrating the importance of contextual variables when evaluating soccer performance. Together, these examples illustrate that soccer performance is complex, and it is futile to reduce its measurement to single variables that can be tested and then summed up. Rather, what might be needed is a global view that sees the contributing aspects in soccer as inseparable elements of a single set that, according to non-linear complexity, are mutually determined (see Di Domenico et al., 2019).

In individual sports, athletes’ career trajectories can be studied by monitoring the development of raw results or by recording more global performance indicators. An individual athlete’s peak performance, and thereby their age at peak performance, can be calculated as the age at their best ever performance during their careers (Haugen et al., 2018). However, in studies that attempt to determine at what average age athletes across different sports reach their career peaks (often called athletes’ peak age), it has been common to consider peak performances such as winning world championships, Olympic medals, and the like (see Allen and Hopkins, 2015; Longo et al., 2016).

In soccer, for obvious reasons, tallying up world championships or similar achievements will not suffice as a measure of individual performance. Soccer is a team sport, and all players of a squad are crowned world champions regardless of their individual contributions (if any). Additionally, the world championship is held every 4 years; thus, a player will have perhaps five or six attempts, at most, to achieve this honour during their career. Becoming a world champion, however, is also dependent on being part of a team that is good enough to win the championship, which means being born in a country that can produce an entire squad of top players. Only eight countries have ever won the FIFA World Cup, and only a handful more have ever reached the final (Britannica, 2020). Furthermore, the timing has to be such that the particular crop of players constituting a squad is not coincident with another, even better, squad.^1^

When attempting to measure “unmeasurable” complex behaviours by selecting sub-variables (sometimes even a single variable), it is a good idea to find some overall proxy against which to validate those measures; this is common practice, especially if any sub-variable (or combination of such) is used for predicting or drawing conclusions about overall complex behaviour. McHale et al. (2012), for example, in an attempt to formulate a soccer player performance rating system, envisaged a hypothetical English National team chosen from the highest ranked players in each position, based on their index scores, and compared the hypothetical team against the actual English team appearing in the first game of the 2010 World Cup. Similarly, McIntosh et al. (2019) validated objective performance indicators by comparing them with subjective ratings of player performance in Australian Rules football.

By validating against such proxies, it is (often implicitly) assumed that the proxy represents the overall complex behaviour in a valid manner, without such assumptions being tested. However, the proxies are intuitively regarded as good measures of the behaviour, which is arguably the case in the mentioned cases of peak sports performance being determined by the winning of world championships and the like.

Our approach was rather the opposite. In the present study, our departure point was a proxy that we believed to be a better representation of the overall complex behaviour that constitutes individual soccer performance. Based on our proxy, we determined age at peak individual soccer performance, and then we compared it with the age at peak performance as reported in other studies. Furthermore, we attempted to validate this against results from studies reporting age at peak performance based on a number of sub-variables that contribute to overall soccer performance. Last, we compared our determined age at peak individual soccer performance with age at peak performance identified in studies involving various other sports.

Previous studies have attempted to determine the peak age of soccer players using different proxies, such as the average age of players at the highest level – notably in the big European leagues (Dendir, 2016) or in the Champions League (Kalén et al., 2019) – or the amount of playing time at the particular level (Dendir, 2016). Other possible proxies for individual performance could be the monetary value of a player (as on Transfermarkt.com) or various rating systems based on the accumulation of counts and ratings of match-related actions, of which Whoscored.com would seem to be the leader (see more details below).

Kalén et al. (2019) argued that the age of peak performance for soccer players was 26.5 years, having increased from 24.9 years in the 1992–1993 season, based on the average age of players in the squads of clubs participating in the UEFA Champions League. Utilising a model that included the average age of the players at the highest club level (the top four leagues in Europe: the Italian Serie A, the German Bundesliga, La Liga in Spain, and the English Premier League), the average playing time in that sample, and those players’ average ratings from Whoscored.com, Dendir (2016) found that soccer players peak at 25–27 years of age, varying with playing position; he stated that his calculated peak age was probably lower and with a narrower age band than in previous reports that suggested peaks in the mid-to-late 20s, which might be due in part to the fact that the sample did not include goalkeepers. Furthermore, as we will discuss, both the above studies included players who were still active at the time; thus, their actual age at peak performance was not yet known.

However, being part of a squad at a high level (as were players in Dendir, 2016 and in Kalén et al., 2019) does not necessarily indicate high performance at that same level. Thus, the average age of such a sample would not be an indication of the peak age of that sample; it would simply indicate the average age. In fact, the average squad age in the top four European leagues is similar to the average squad age in any European league. The average age of European soccer players is currently 26 years. The average squad ages across European leagues vary from 24.3 (Slovakia) to 28 years (Turkey), with ages in the top five leagues varying from 25.6 (France) to 26.9 years (Italy; Poli et al., 2019), probably reflecting midpoints of average-length careers.

WhoScored (2020) ratings, as indicated above, are promising as indicators of individual soccer performance, but they have notable methodological shortcomings. The ratings are somewhat condensed around the average (which Who Scored defines as six points). Most importantly, however, ratings are relative to playing level, reflecting performances relative to teammates and opposing players at that same level. Thus, they cannot be meaningfully compared across levels of performance. For example, the average score for the top 50 players in the German Bundesliga in the 2019–2020 season was 7.2 points, quite similar to 7.1 points in the Zweite Bundesliga (the second-highest-rated German league; the league Bundesliga teams are relegated to or promoted from). This fact might not constitute a problem when comparing players across similar levels, such as the top five leagues (which WhoScored does). However, it introduces problems when, for example, comparing a player with himself across a career when that career includes playing at different performance levels.^2^ Knowing that the best players in the lower leagues will migrate to the top five leagues and that many top players in fact started their careers in lower-ranked leagues, a significant share of players would not be comparable to themselves over time in terms of performance if one relies on WhoScored ratings, and for some, it would not even be possible to find complete records.

Furthermore, the WhoScored rating system dates back only some 10 years; thus, performances predating this can neither be compared within nor across players. Adding the effect of a (assumedly) continuously updated algorithm, one could argue that ratings are not comparable over time, at least not over longer periods.

In the present study, as mentioned, we introduce the ages at nomination of major player of the year award recipients as a performance proxy, with the argument that this variable, in fact, is the closest one can come in soccer, being a team sport, to counting championship medals within individual sports (see Allen and Hopkins, 2015; Longo et al., 2016).

The most renowned player of the year awards are the Ballon d’Or, the FIFA Player of the Year award, and the combined FIFA Ballon d’Or and the UEFA Player of the year awards. The Ballon d’Or is “one of the oldest and generally regarded as the most prestigious individual award for football players, and it has been awarded since 1956” (Ballon d’Or, 2020). “Doing well in the Ballon d’Or election is an invaluable stamp of approval that authenticates and personalizes achievement and confers power and status to those players who place near the top” (Anderson et al., 2020, p. 94). There are no exact performance measures that qualify players for the awards. The Ballon d’Or prize organiser, France Football, provides a shortlist of 30 players. From that list, international journalists and the coaches and captains of the national teams under FIFA’s jurisdiction are eligible to vote for five players they deem to have performed the best in the previous calendar year (Ballon d’Or, 2020). The jury evaluates the players according to several subjective criteria, including on-field performance as well as a player behaviour on and off the field. Voters are instructed to consider a player’s individual and team performances during the previous 12 months, including championships won, skill and fair play on the field, career accomplishments, and, interestingly, a player’s appeal (Anderson et al., 2020).

Coupe et al. (2018) investigated biases and strategic behaviour related to performance evaluations in jury voting for the FIFA Ballon d’Or award. They found that national biases are substantial, with jury members disproportionately voting for candidates from their own countries, national teams, continents, and leagues. However, the impact of such biases on the total number of votes a candidate receives is fairly limited and hence is likely to affect the outcome of the Ballon d’Or only on rare occasions (Coupe et al., 2018). Regarding strategic voting, jury candidates are actually more, rather than less, likely to vote for their main competitor (Coupe et al., 2018). Coupe et al. (2018) investigated jury voting for the nominated players, but, to the best of our knowledge, no studies have looked into the nomination process itself.

Nominations for player-of-the-year awards seem to be based entirely on subjective evaluations by soccer experts in, for example, France Football for Ballon d’Or. Laurence (2011) argued that when measured by the WhoScored rating, six players other than those shortlisted for FIFA Ballon d’Or should have been included, and five others should have been excluded. Regardless of such inconsistencies, the Ballon d’Or and the other major awards are widely recognised, and there is no doubt that being nominated for one of them indicates a career peak for a player.

The purpose of the present study was to

1. Determine the age at individual peak performance for soccer players using our proxy.
2. Discuss how the peak age changes with operationalisation of the dependent (proxy) variable.
3. Discuss our proxy relative to other (proxy) variables used in previously published studies.
4. Discuss the age at peak performance based on our proxy variable relative to peak performance based on contributing (sub-)variables.

## Materials and Methods

### Player Sample and Procedure

The present data comprised the soccer players nominated for awards each year from 1956 to 2019, and they provided us with the opportunity to record each player’s (often multiple) career peaks in a manner similar to that used in studies determining age at peak performance in other sports (Allen and Hopkins, 2015; Longo et al., 2016). From these peaks, each player’s age at peak performance was determined according to several different operationalisations of the peak (see below). Furthermore, a detailed timeline of players’ average ages at nomination was established from the first-ever Ballon d’Or in 1956 up to and including major awards for 2019.

Different operationalisations of the proxy are interesting and relevant for different reasons. The *age at player’s first nomination* is relevant in a developmental context because it provides information on how early in a career it is possible to perform at the very top level of international soccer (viz the 10,000-h/10-year rule; Ericsson and Lehmann, 1996). The *average age at nomination for awards* indicates the typical age at which players perform at their best, and it can be compared with similar ages in other sports. Last, the *last time a player was nominated for an award* indicates the age up to which top players, on average, can expect to maintain their highest performance level, which may be of importance in contract negotiations and when trading players (see Dendir, 2016).

All players nominated (not merely those receiving awards) for the various player of the year awards during the period 1956–2019 were included (*N* = 1,981). Nomination lists for player-of-the-year awards, including nominees for positional awards, were collected for Ballon d’Or (Rec.Sport.Soccer Statistics Foundation, 2020a), FIFA World Player of the Year (Rec.Sport.Soccer Statistics Foundation, 2020b) and The Best Fifa Men’s Player (Wikipedia, 2020). The main data source was the annual football award, Ballon d’Or, presented by France Football and awarded during the periods 1956–2009 (*N* = 1,571) and 2016–2019 (*N* = 123). Additionally, nominations were collected from the merged FIFA and Ballon d’Or for 2010–2015 (*N* = 125), the UEFA Men’s Player of the Year from 2011 to 2019 (*N* = 138), the FIFA World Player of the Year from 1991 to 2009 (*N* = 19), and the Best FIFA Men’s Player from 2017 to 2019 (*N* = 5).

When a player was nominated for several awards in the same season, only one of them was counted for that season. This total sample of nominated players was used to identify each player’s age at nomination, and this was used to calculate the *average age at nomination, average age of a player’s first nomination*, and *average age at a player’s last-ever nomination*.

From this total sample of nominated players, a subsample was extracted to identify each player’s career peaks. Players who had not yet finished their careers were excluded, resulting in a retired player subsample (*n* = 685). The latter sample consisted of 661 nominations for Ballon d’Or, six nominations for Ballon d’Or/FIFA, eight nominations for the Best FIFA Men’s Player, and eight nominations for the UEFA Men’s Player of the Year.

In addition, from the player-of-the-year data, players’ positions on the pitch were recorded as registered in the nomination for the award (when applicable) and further verified at Transfermarkt.com. Positions were further re-categorised into four general positions: goalkeeper, defender, midfielder, and forward.

Players’ ages were recorded (as in Dendir, 2016) as of January 15, approximately the mid-season mark for a typical league measured on a continuous scale. For instance, a player born on October 26, 1984 was 28.2 years of age in the 2012–13 season.

Some players had been nominated more than once. In these cases, both the player’s average age over several nominations (a single player can represent multiple cases) and the player’s age at first or last-ever nomination (each player is represented only once) was calculated. In order to create a timeline of the average ages of the nominated players, we calculated their average ages for each year from 1956 to 2019.

### Sources: Major Individual Soccer Awards

From its inception in 1956 until 2006, the winner of the Ballon d’Or award was chosen by football journalists only. Since 2007, coaches and captains of national teams have been eligible to vote. Originally, it was an award for players from Europe, but in 1995 the Ballon d’Or was expanded to include all players of any origin who had been active in European clubs and, subsequently, the award became global in 2007.

The FIFA World Player of the Year award was presented annually by FIFA from 1991 to 2009. The award was merged with the Ballon d’Or from 2010 to 2015 and re-established as the FIFA Best Men’s Player of the Year award in 2016. Coaches and captains of international teams, as well as international media representatives, decide the nominations for this award.

The UEFA Club Footballer of the Year was presented annually by UEFA from the 1997–98 season to the 2010–11 season, when it was replaced by the UEFA Best Player in Europe award, originally established to revive the Ballon d’Or, which had merged with the FIFA World Player of the Year in 2010. The recipients of the UEFA Men’s Player of the Year award are selected from among players in the European leagues. In the beginning, this award was decided by 53 sports journalists (later expanded to 55) representing each of the UEFA national associations. We have not succeeded in retrieving shortlists of all nominees, only listings of winners of the positional awards during the period 1997–2010.

### Ethics

The present study was based on publicly available, non-sensitive data from the above-mentioned websites. Thus, ethics approval was not required per applicable institutional and national guidelines and regulations.

### Statistical Analysis

Kolmogorov-Smirnov tests (KS), histograms, and Q-Q plots were applied to examine normality assumptions of the variables’ statistical distributions, and they indicated a normal distribution (KS test = 0.015, *p* > 0.20). Distribution of nominations across playing positions was examined with Chi-square tests (*χ*^2^) against an even distribution. Potential position-related differences in age at nomination were examined by one-way ANOVAs with partial eta squared (*ղ*^2^_p_) as the indicator of effect size, interpreted as small effect: 0.01, medium effect: 0.06, and large effect: 0.14 (Cohen, 1988; Richardson, 2011). Position-related differences were further examined with Bonferroni-corrected *post hoc* analysis with Cohens *d* applied as a measure of effect size, in which 0.2, 0.5, and 0.8 were considered small, moderate, and large, respectively (Cohen, 1988; Lakens, 2013). Pearson product-moment correlations were used to examine the relation between year of nomination and average age of nomination at various timepoints. Predictive Analytics Software (PASW, IBM, United States; previously SPSS) version 27.0.0.0 was used for all statistical procedures, with *p* < 0.05 as the statistical significance criterion.

## Results

### Total Sample of Nominated Players

**Figure 1:**
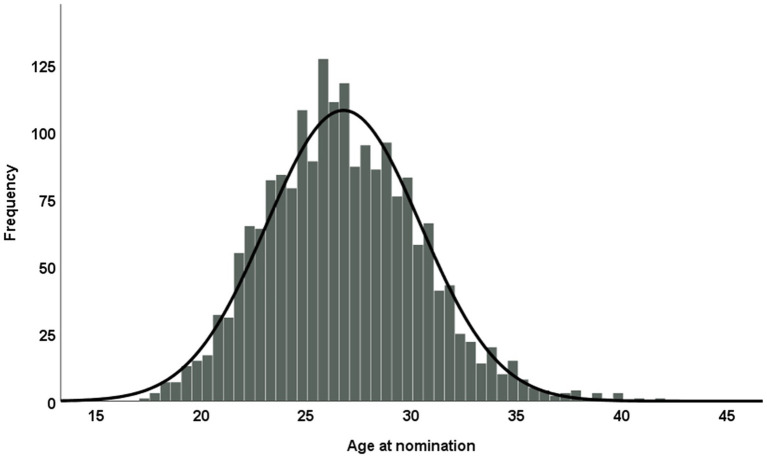
Frequency of nominations at different ages for the total sample.

In the overall sample of 1,981 nominations, 9.2% were goalkeepers; 15.2% were defenders; 32.2% were midfielders, and 43.5% were forwards. The distribution of nominations across positions differed significantly from an even distribution (*χ*^2^ ≥ 176, *p* < 0.001), with defenders being underrepresented relative to the number of defending positions and attackers being somewhat overrepresented. The age at nomination was normally distributed for the total sample of nominations (see Figure 1; the mean age of players in the total sample was 26.75 years, with a SD of 3.66 years).

### Age at Nomination

The mean age at nomination, as can be seen in Table 1, varied across playing positions. An initial one-way ANOVA indicated significant differences {*F* [1,3] = 65.77, *ղ*^2^_p_ = 0.09 [95% CI (0.07; 0.12)], *p* < 0.001} across playing positions. Further *post hoc* analysis with Bonferroni correction indeed confirmed that age at nomination was significantly different between all positions {*d* ≥ 0.50 [95% CI (0.31; 0.69)], *p* < 0.001} except between midfielders and forwards {*d* = 0.14 [95% CI (0.04; 0.24)], *p* = 0.071}.

**Table 1:** Age at nomination across playing positions for the total sample.

Position	*n* (% of total)	Average age	First nomination	Last nomination
		Mean (SD)	Mean (SD)	Mean (SD)
Goalkeepers	182 (9.19)	29.64 (4.01)	28.24 (3.60)	30.43 (4.21)
Defenders	301 (15.19)	27.82 (3.38)	26.70 (3.19)	28.36 (3.38)
Midfielders	637 (32.16)	26.46 (3.42)	25.30 (3.26)	27.39 (3.44)
Forwards	861 (43.46)	25.99 (3.47)	25.08 (3.48)	27.28 (3.45)
Total	1981 (100.00)	26.76 (3.66)	25.73 (3.51)	27.80 (3.63)

### Age at First Nomination

The mean age at first nomination (Table 1) also varied across playing positions. An initial one-way ANOVA indicated significant differences {*F* [1,3] = 24.34, *ղ*^2^_p_ = 0.08 [95% CI (0.05; 0.12)], *p* < 0.001}. Further *post hoc* analysis with Bonferroni correction confirmed that age at nomination was significantly different between all positions {*d* ≥ 0.46 [95% CI (0.18; 0.74)], *p* < 0.001} except between midfielders and forwards {*d* = 0.09 [95% CI (−0.07; 0.25)], *p* = 0.29}.

### Age at Last-Ever Nomination

One-way ANOVA indicated significant differences {*F* [1,3] = 19.73, *ղ*^2^_p_ = 0.07 [95% CI (0.04; 0.10)], *p* < 0.001} between playing positions for mean age at last nomination (Table 1). *Post hoc* analysis with Bonferroni correction indicated that age at last nomination was significantly different between all positions {*d* ≥ 0.56 [95% CI (0.28; 0.84)], *p* < 0.001} except between midfielders and forwards {*d* = 0.05 [95% CI (−0.11; 0.21)], *p* = 0.55}.

### Age at Nomination Over the Period 1956–2019

**Figure 2:**
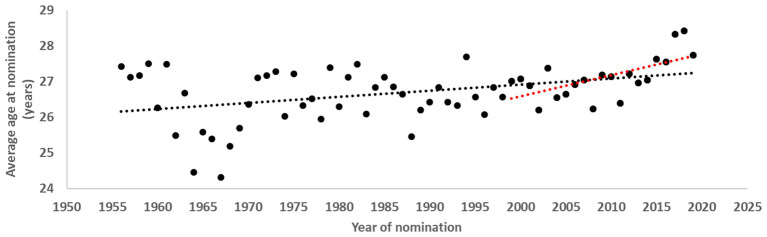
Development of mean age at nomination from 1956 to 2019. Black dotted line indicates trendline for the entire period; red dotted line indicates development in the past two decades (1999–2019).

As can be seen in Figure 2, the mean age at nomination for each year increased, culminating with a mean age at nomination of 28.4 years in 2018. From 1956 to about 1967, the average age at nomination dropped from about 27.5 to 24.5 years before increasing again to reach the initial 1956 values in 1973. During 1974–2014, a period of rather stable ages at nomination was followed by a considerable increase of 1.3 years from 2014 to 2018. Thus, a significant relationship was found between year of nomination and average age at nomination during the period 1956–2019 (*r* = 0.41, *p* < 0.01). For the most recent 2 decades (1999–2019), an even stronger statistical relationship was found between year of nomination and average age at nomination (*r* = 0.63, *p* < 0.01).

### Subsample: Retired Players

**Figure 3:**
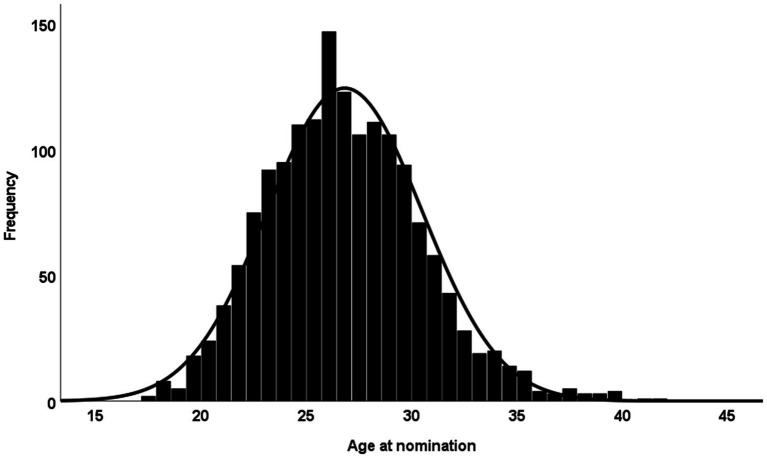
Frequency of nominations at different ages for retired players’ last nominations.

The subsample of confirmed retired players (*n* = 685) consisted of 8.6% goalkeepers, 17.0% defenders, 32.6% midfielders, and 41.8% forwards. The number of nominations was normally distributed by age; the most frequent age at nomination was from 26 to 27 years (see Figure 3).

### Age at Nomination for Retired Players

The mean age at nomination for retired players (Table 2) also varied across playing positions. An initial one-way ANOVA indicated significant differences {*F* [1,3] = 63.56, *ղ*^2^_p_ = 0.11 [95% CI (0.08; 0.13)], *p* < 0.001} across playing positions. Further *post hoc* analysis with Bonferroni correction confirmed that age of nomination was significantly different between all positions {*d* ≥ 0.32 [95% CI (0.17; 0.47)], *p* < 0.001} except between midfielders and forwards {*d* = 0.15 [95% CI (0.04; 0.27)], *p* = 0.055}.

**Table 2:** Age at nominations across playing positions for retired players.

Position	*n* (% of total)	Average age^1^	First nomination	Last nomination
		Mean (SD)	Mean (SD)	Mean (SD)
Goalkeepers	60 (8.8)	30.28 (3.99)	28.57 (3.46)	31.03 (4.29)
Defenders	113 (16.5)	27.65 (3.41)	26.71 (3.26)	28.41 (3.33)
Midfielders	223 (32.6)	26.55 (3.43)	25.39 (3.32)	27.65 (3.44)
Forwards	289 (42.2)	26.03 (3.43)	25.13 (3.44)	27.94 (3.64)
Total	685 (100.00)	26.81 (3.68)	25.78 (3.52)	27.91 (3.61)

1 All nominations (*n* = 1609).

### Age at First Nomination for Retired Players

The mean age at the first year of nomination for retired players was 25.78, with SD at 3.68 (see Table 2). One-way ANOVA indicated a significant difference in age at first nomination across playing positions {*F* [1, 3] = 21.13, *ղ*^2^_p_ = 0.09 [95% CI (0.05; 0.12)], *p* < 0.001}. *Post hoc* analysis indicated that age at nomination was significantly different between all positions {*d* ≥ 0.40 [95% CI (0.17; 0.63)], *p* < 0.005} except between midfielders and forwards {*d* = 0.08 [95% CI (−0.10; 0.25)], *p* = 0.40}.

### Age at Last-Ever Nomination for Retired Players

The mean age at last year of nomination for retired players was 27.94, with SD at 3.64 (Table 2). A one-way ANOVA indicated a significant difference in age at last nomination across playing positions [*F* [1, 3] = 19.59, *ղ*^2^_p_ = 0.08 [95% CI (0.04; 0.12)], *p* < 0.001], and a *post hoc* analysis indicated that goalkeepers were significantly older at year of last nomination compared to players in other positions {*d* ≥ 0.71 [95% CI (0.39; 1.03)], *p* < 0.001} and that defenders were significantly older compared to forwards {*d* ≥ 0.31 [95% CI (0.10; 0.53)], *p* < 0.01}. No further position-related differences in age at last year of nomination for the retired players was found {*d* ≤ 0.22 [95% CI (−0.003; 0.451)], *p* > 0.05}.

## Discussion

The average age at nomination for major individual awards was 26.8 years in the total sample. There was considerable variation across playing positions, with forwards and midfielders peaking earlier than defenders, 26 and 26.5 years vs. 27.8 years, respectively, and goalkeepers peaking particularly late, at 29.6 years. There were, however, interesting variations across the different operationalisations of the proxy.

The average age at a player’s first nomination for major individual awards was 25.7 years. For this variable as well, there were notable differences across playing positions. Forwards and midfielders were youngest when first nominated, at just above 25 years; defenders were closer to 27 years, whereas goalkeepers were oldest by some margin, receiving their first-ever nomination on average at 28 years of age.

It could be argued that the average age at player’s first nomination indicates how long it takes to consistently perform among the very best in the game. This would occur some 7–8 years after entering that level for outfield players, and one extra year for goalkeepers. This first peak age is similar to those estimated by Dendir (2016) and Kalén et al. (2019), and it seems to correspond well with findings in top-performing groups from different walks of life, which formed the basis for the 10-year rule for developing expertise within a domain (Ericsson and Lehmann, 1996). In two oft-cited studies, Simonton (1991a) showed that composers of classical music produced their first successful composition some 9 years after composing their first piece of music. Similarly, scientists produced their best work some 10 years after they produced their first work (Simonton, 1991b). Of course, scientists’ and composers’ careers are not directly comparable to soccer players’, whose careers are much shorter. However, the trend is compelling and has since been replicated for many other domains (see Ericsson and Lehmann, 1996).

The average age at nomination for players in our total sample (thus their average age at peak performance) is slightly below peak ages in other team-ball sports. The age at peak performance of athletes in field hockey, basketball, water polo, volleyball, handball, and beach volleyball ranges from 27 to 30.5 years (Longo et al., 2016). These findings are supported by Brander et al. (2014), who reported the peak age of ice hockey players in the NHL to be 28 years (forwards 27.7 and defensemen 28.2). In line with this, Vaci et al. (2019) reported NBA basketball players to peak around 28–29 years of age. In our sample of retired players, the peak age is more similar to those sports, albeit it is in the nether regions of the age span. Also, the variations across playing positions are similar, with defensive players peaking a year or two later than those in attacking positions.

The average age at nomination has increased from 1956 to 2019, the increase being particularly strong in the last decade or so. This finding is similar to that of Kalén et al. (2019), who found that soccer players currently at the highest level are on average older compared to those who played some 25 years ago. In the present sample, the average age of nominated players increased by almost 3 years compared to the early 1960s. In fact, 2 out of the 3 years of increase occurred in the last decade, with the average age at nomination culminating in 2018–2019 at 28.4 years.

In the total sample, the average age at players’ last nomination was 28 years, with similar variations across playing positions as described for the total sample. It can be argued that this constitutes the player’s last career peak; thus, it indicates the point in time when a player’s physical capacity is reduced enough that it is difficult to maintain the highest performance level, even when excelling in other variables such as technical, perceptual, or tactical skills. This inflection point was identified by Vaci et al. (2019) to occur on average at 27–28 years of age for basketball players. Our argument would be supported by the fact that defenders, playing in the position that includes the least and the slowest running, maintain their performance levels on average a year longer. Even more so, goalkeepers, having completely different physical requirements that include limited amounts of running, continue to be nominated on average 3 years longer compared to forwards and midfielders.

As discussed in the introduction of the present paper, calculating the average age of players participating at a certain level (in the present case, those who have been nominated for major individual awards) does not provide information on individual players’ peaks. Thus, we cannot establish whether they actually peak at this age. Therefore, in order to examine individual players’ actual peaks, the subsample of retired players was consulted.

Among retired players, the average age at nomination was higher. More specifically, it was 27.9 years, varying from 27.3 to 27.5 years, for forwards and midfielders, respectively, 28.6 years for defenders and up to 31 years for goalkeepers. This operationalisation (last-ever nomination) could be argued to be a more accurate measure of a player’s age at peak performance, as each player’s actual peak was known. In the total sample, however, similarly to Dendir (2016) and Kalén et al. (2019), the average represents players’ average age of nomination thus far in their careers.

In fact, we had expected that the average age at nomination would be higher in the subsample of retired players. The fact that the average was similar to that of the total sample indicates that our suggested peak age may be somewhat underestimated. That the average peak age at nomination is similar in a sample of players including many who are still active compared to a sample of retired players suggests that ultimately the peak age will increase in the former sample. Judging by the increase in the average age at nominations for awards over time (depicted in Figure 2) of 2 years during the last decade, as well as the 1.6-year increase since 1992–1993 in the average age of players in the Champions League (Kalén et al., 2019), it is not unreasonable to speculate that the age of peak individual soccer performance for players at the highest possible level lies closer to 29 years and that players may, on average, continue to be nominated for best player awards long after they have turned 30, especially when they play in defensive positions. Thus, defenders may continue to perform at their highest levels well into their 30s, and goalkeepers, with their special and less physically demanding skillset, may sustain careers at the top level up to 40 years of age. The latter assumption is supported by the fact that goalkeepers in the present sample had their first peak at age 28 and their last when they were over 30 years old, which is within the overall trend of increasing age.

The age at peak soccer performance for an individual player would then occur some 10 years after the age at which they entered the top level, which for most players is just under 20. Thus, the 10-year rule (Ericsson and Lehmann, 1996) holds even for top-level soccer players. Their decline, however, is rather steep compared with those of experts within other domains, as soccer players on average experience their last peak a year or so after their average peak and only 2–3 years after their first peak, indicating a biphasic career trajectory. This would be due to physical decline (see Guillaume et al., 2011 and Berthelot et al., 2012) that can no longer be compensated for by superior skills. However, players’ careers may last much longer, and even after they stop being nominated for awards, players continue to play at the highest level for several years.

When individual athletes win world championships or Olympic medals, it is recognised as a clear career peak (Allen and Hopkins, 2015; Longo et al., 2016), indicating an athlete’s peak age in that sport. We would argue that being nominated for major individual awards constitutes a similar achievement among soccer players as well as, probably, athletes in other team sports, indicating that age at nomination for awards is a good proxy for peak performance of individual players.

WhoScored ratings, for reasons mentioned earlier, cannot be used for comparisons of individual players’ performances across different performance levels. Still, if the goal is to distinguish between performances at the absolute top level, it would be reasonable to compare across the top five leagues. Out of 427 nominations for player of the year since 2005, only 16 (Ballon d’Or, 2020) were for players outside the Big Five.

As suggested above, soccer may value and emphasise attacking players over defenders. This might be due to the narrative of TV productions focusing mainly on goal scorers (Anderson et al., 2020). Although popularity and public voting has been included to a certain extent in the criteria for the awards, performance as judged by soccer experts has always been the main evaluation criterion. Furthermore, popularity and performance might be quite entangled. Yucesoy and Barabási (2016) showed, for example, how a predictive model comprising data on a tennis player’s performance in tournaments can accurately predict the athlete’s popularity, and it is argued that in most areas of human achievement, exceptional visibility may be rooted in detectable performance measures.

While nominations have, at least in the most recent years, been somewhat more balanced with respect to playing positions, still, in our total sample, players occupying attacking and midfield positions are overrepresented at around 70% of the nominated players. At the same time, their relative representation on the field, independent of the playing system (e.g., 4-3-3, 4-4-2) was around 55%.

Dendir (2016) found the peak age of soccer players to be from 25 to 27 years of age, depending on playing position, with forwards peaking earliest at 25, defenders last at 27, and midfielders somewhere from 25 to 27, depending on which model was used for calculation. From the work of Dendir (2016), it is reasonable to estimate that the peak age of outfield soccer players lies somewhere around 26 years. Goalkeepers were excluded from sample of Dendir (2016) due to their rather unique skillset, thus calculating a total average for soccer players was not possible. If we exclude goalkeepers from our sample of retired players, individual peak performance is 27.8 years. Thus, the reported peak age of sample of Dendir (2016) was considerably lower than in the present dataset, probably reflecting the fact that it takes somewhat longer to reach the absolute top level compared to having played in the Big Four leagues. Also, Kalén et al. (2019) reported an average peak age of 25.8 years for players in the Champions League, considerably lower than that of the present sample.

The age distribution in sample of players of Dendir (2016), hence the average age of players who had played a minimum of three games in one of the top four European leagues, was normal around the average of 26–27 years (indicating a bimodal career trend), as was the case for the average age at peak minutes played. However, the picture was more complex for the WhoScored ratings. Especially with defenders, these ratings were unrelated to the former two variables, and for forwards an additional peak was evident at around 37 years.

Kalén et al. (2019) reported an average age for players in the UEFA Champions League (more specifically, players who were members of squads on participating teams) of 26.5 years. Furthermore, they reported an increase of 1.6 years in the average age from the 1992 to 1993 season.

Our finding of differences in age at peak performance for different playing positions (similar to both Dendir, 2016 and Kalén et al., 2019) seems to be related to physical and technical requirements for the various playing positions. Also similar to the present results, Kalén et al. (2019) concluded that goalkeepers and centre backs peak later and may maintain their peak performance until about 31 years of age. Fitness profiling in soccer identifies attackers as the quickest players (Sporis et al., 2009). Specialised sprinters peak at 25–26 years of age (Haugen et al., 2018), and it seems that soccer players’ sprint times can be maintained until 28 years of age (Haugen et al., 2020). The average age at nomination for forwards in our total sample was 26 years, coinciding with peak sprint times, and it was similarly around 26.5 years for midfielders. Also, the decline in sprint performance is rather coincident with age at last nomination in awards for midfielders and forwards. It should be kept in mind, however, that the possible underestimation of peak age indicates that while sprint times are important, many additional variables contribute to the mix that is soccer performance, not the least of which are ball and tactical skills (Sal de Rellán-Guerra et al., 2019); thus, players may continue to be nominated well after their peaks. The oldest nominated forward of all time was Stanley Matthews at 42 years in 1956. More recently, Zlatan Ibrahimović was nominated for the FIFA Best Player award at the age of 35.6 years in 2017 (and still, in 2020, he continues to score goals in the Italian Serie A).

As discussed in the introduction, endurance performance in specialised endurance athletes generally peaks later as the duration of the race increases, up to the late 30s in races of long duration (Longo et al., 2016). This also seems to be the age Botek et al. (2016) recognised as the inflection point of endurance performance, while Tønnessen et al. (2013) reported 18-year-old players to have higher maximal oxygen uptake than older players. Results regarding peak endurance capacity are somewhat equivocal, and as for sprint capacity, it seems that endurance sufficient to elicit nomination for player of the year awards can be maintained at a late age. Midfielders generally have the highest maximal oxygen uptake among players in all positions (Sporis et al., 2009; Tønnessen et al., 2013), and from our data, we identified an average age of peak performance for the total sample of midfielders to be 26.46 (27.53 for retired last nomination). Our estimated ages at peak performance relative to age variations in endurance capacity seem to reflect the findings on sprint performance, and they indicate that a decrease in endurance can be compensated for by improved performance regarding technical and tactical skills (Sal de Rellán-Guerra et al., 2019). The midfielder Lothar Matthäus was nominated for Ballon d’Or in 1999 at the age of 37.8 years, being to date the oldest nominated midfielder.

In summary, the age at last nomination in our sample of retired players corresponds well with peak sprint performance among both specialised athletes and soccer players, while for endurance, the literature on peak performance is equivocal. However, the age range of nominated players supports the findings of Sal de Rellán-Guerra et al. (2019) and Wilson et al. (2017) that although players may be past their peaks as to several physiological attributes, soccer performance can be maintained at a high level by compensating with other non-physiological skills that are less subject to decline after the age of 25.

We must again remind the reader that the results are based on data for the proxy *nomination for major international individual awards* and that these do not directly measure performance. Thus, we cannot directly establish age of peak performance. However, as we have argued, no other variable can directly measure individual soccer performance on an overall level. Furthermore, the results cannot necessarily be generalised to populations other than the absolute top players comprising the present sample.

## Conclusion

Age at peak individual performance, when estimated based on our proxy, is higher compared with previous studies, which can partially be accounted for by differences between the various proxies and their operationalisations. As in previous studies, average age at peak performance varied considerably across the different operationalisations, thus supporting the notion that players have rather distinct skillsets across playing positions. Furthermore, the age is higher than peak ages for the many physiological variables that contribute to soccer performance, indicating that soccer skills are more important than physique. Players in defensive positions, goalkeepers in particular, were nominated for awards at more advanced ages compared with players in attacking positions.

We would argue that compared with proxies used in previous studies, our proxy is most similar to those most often used in studies on age of peak performance in individual sports, namely the age when winning world championships or Olympic medals. This would make ours a good proxy, and our average age a decent estimate of the age at peak individual soccer performance. Furthermore, and perhaps more importantly, our proxy, as well as other proxies, may be used to validate the myriad of variables measured in studies of soccer skills to enable comparisons or predictions of individual soccer performance.

In fact, we would argue that our approach using proxies to unearth information about hidden features of otherwise immeasurable complex performance, depending on a host of variables acting in concert, is viable. Furthermore, we would argue that such proxies may be used for validating single variables or combinations of limited numbers of variables that are touted as being explanatory of complex behavior.

## Data Availability Statement

The original contributions presented in the study are included in the article/supplementary material, further inquiries can be directed to the corresponding author.

## Author Contributions

GO and AVP conceived the idea, and all three co-authors designed the study. GO collected the data together with AVP and analysed them together with HL. GO wrote the first draft of the manuscript. All three co-authors contributed substantially to the final version of the manuscript and approved it for publication.

### Conflict of Interest

The authors declare that the research was conducted in the absence of any commercial or financial relationships that could be construed as a potential conflict of interest.

## Footnotes

^1^Many of the best players of all time, according to the website FourFourTwo (2020), were not part of teams that won the World Cup, including Eusebio, Lev Yashin, Paolo Maldini, Zico, Franco Baresi, George Best, Marco van Basten, Michel Platini, Ferenc Puskas, Alfredo Di Stefano, Johan Cruyff, Lionel Messi, and Cristiano Ronaldo (although a few of these have won the UEFA European Championship), to mention but a few household names. Additionally, some will remember the team that is often described as the best team to never win the WC, Brazil of 1982, boasting players such as Zico, Socrates, Junior, and Eder.

^2^For illustration, the European Golden Shoe is awarded by European Sports Media (ESM) to the player scoring the most goals in a European league each season (European Sports Media, 2020). In order to fairly compare performances across leagues, from 1996 onwards a points system has been applied in which goals scored in the top five leagues are multiplied by a factor of 2, whereas goals scored in leagues ranked 6th through 21st are multiplied by 1.5. Any goals scored in any of the remaining leagues are multiplied by 1.0 (European Sports Media, 2020). Thus, a player would need to score twice as many goals in a lower-ranked league compared to the top five in order to win the Golden Shoe. In the 2019–2020 season, Erling Braut Haaland of Borussia Dortmund accumulated a total of 50 goal points from 29 goals, 16 for Red Bull Salzburg (multiplied by 1.5) and 13 goals for Dortmund (multiplied by 2). In the same season, Lionel Messi accumulated 50 goal points for his 25 goals for Barcelona.
